# Supplementary material for: Gas-Phase Structure of 3,7,9-tris(trifluoromethylsulfonyl)-3,7,9-triazabicyclo[3.3.1]nonane by GED and Theoretical Calculations
Source: Molecules. 2023 May 6;28(9):3933. doi: 10.3390/molecules28093933 (PMC10179815; doi:10.3390/molecules28093933)
Supplement: Supplementary file 1 [file molecules-28-03933-s001.zip › molecules-2361084-supplementary.pdf]

molecules

Article

Gas-Phase Structure of 3,7,9-tris(trifluoromethylsulfonyl)-3,7,9-triazabicyclo[3.3.1]nonane by GED and Theoretical Calculations

Bagrat A. Shainyan [1,*], Alexey V. Eroshin [2], Valeriya A. Mukhina [2] and Sergey A. Shlykov [2,*]

[1] A. E. Favorsky Irkutsk Institute of Chemistry, Siberian Division of the Russian Academy of Sciences, 1 Favorsky Street, 664033 Irkutsk, Russia
[2] Department of Physical and Colloidal Chemistry, Ivanovo State University of Chemistry and Technology, Sheremetevkiy Ave, 7, 153000 Ivanovo, Russia; alexey.yeroshin@gmail.com (A.V.E.); lera.muhina2011@yandex.ru (V.A.M.)
* Correspondence: bagrat@irioch.irk.ru (B.A.S.); shlykov@isuct.ru (S.A.S.)

Content

Cartesian coordinates of 3,7,9-tri(trifluoromethylsulfonil)-3,7,9-triazabicyclo[3.3.1]nonane calculated at B3LYP/cc-pVTZ level 2

Cartesian coordinates of 3,7,9-tri(trifluoromethylsulfonil)-3,7,9-triazabicyclo[3.3.1]nonane calculated at B3LYP/aug-cc-pVTZ level 8

Cartesian coordinates of 3,7,9-tri(trifluoromethylsulfonil)-3,7,9-triazabicyclo[3.3.1]nonane calculated at M06-2X/cc-pVTZ level 14

Cartesian coordinates of 3,7,9-tri(trifluoromethylsulfonil)-3,7,9-triazabicyclo[3.3.1]nonane calculated at M06-2X/aug-cc-pVTZ level 20

Equation S1. Disagreement factor being minimized at least-squares procedure of the GED data. 26

Equation S2. Molecular scattering intensities 26

Table S1. Selected geometric parameters (Å and degrees) of 1 according to QC calculations. 27

Table S2. Disagreement factors R_f and refined conformers contribution X evaluated from least-squares refinement using the UNEX program. The $3\sigma_{LS}$ quantities were adopted as error limits 31

Table S3. Dependence of agreement factor on the conformers ratio with a step of 2% in the region of minimal R-factors 32

Cartesian coordinates of 3,7,9-tri(trifluoromethylsulfonil)-3,7,9-triazabicyclo[3.3.1]nonane calculated at B3LYP/cc-pVTZ level

1-c-out-2-c-out			
7	0.200344000	1.777128000	0.000000000
6	0.754979000	1.249582000	1.253910000
6	0.798839000	-0.284229000	1.222264000
6	0.798839000	-0.284229000	-1.222264000
6	-0.562405000	-0.989929000	1.255113000
7	-1.295257000	-0.768125000	0.000000000
6	-0.562405000	-0.989929000	-1.255113000
7	1.500782000	-0.715048000	0.000000000
16	3.057600000	-1.226794000	0.000000000
16	-0.671292000	3.171622000	0.000000000
6	2.878161000	-3.099679000	0.000000000
6	0.627673000	4.538280000	0.000000000
8	3.660148000	-0.903920000	-1.272723000
8	3.660148000	-0.903920000	1.272723000
8	-1.342193000	3.290973000	-1.271740000
8	-1.342193000	3.290973000	1.271740000
9	2.203808000	-3.488285000	-1.085743000
9	4.076934000	-3.664295000	0.000000000
9	2.203808000	-3.488285000	1.085743000
9	0.040952000	5.726832000	0.000000000
9	1.400261000	4.424910000	-1.085510000
9	1.400261000	4.424910000	1.085510000
16	-2.941547000	-0.779551000	0.000000000
8	-3.401518000	-0.277690000	-1.271670000
8	-3.401518000	-0.277690000	1.271670000
6	-3.401631000	-2.609556000	0.000000000
9	-2.892244000	-3.203172000	1.085374000
9	-2.892244000	-3.203172000	-1.085374000
9	-4.719157000	-2.750031000	0.000000000
6	0.754979000	1.249582000	-1.253910000
1	-0.386679000	-2.057328000	-1.416581000
1	-1.164759000	-0.607792000	-2.074613000
1	-0.386679000	-2.057328000	1.416581000
1	-1.164759000	-0.607792000	2.074613000
1	1.370983000	-0.615036000	-2.085422000
1	1.370983000	-0.615036000	2.085422000
1	0.133500000	1.594097000	2.075885000
1	1.775046000	1.613068000	1.408542000

1	0.133500000	1.594097000	-2.075885000
1	1.775046000	1.613068000	-1.408542000

1-c-in-2-c-out			
7	-0.931672000	1.094970000	0.015117000
16	-2.518787000	1.534193000	0.023715000
8	-3.079536000	1.186092000	1.307317000
8	-3.106456000	1.140885000	-1.234542000
6	-2.507803000	3.421996000	-0.009714000
9	-1.854025000	3.888104000	1.059061000
9	-1.883037000	3.850354000	-1.111118000
9	-3.750537000	3.882115000	-0.001178000
6	-0.174348000	1.132889000	-1.245466000
1	-0.852512000	0.891113000	-2.059131000
1	0.241317000	2.129468000	-1.420004000
6	0.994244000	0.138968000	-1.209142000
7	1.794168000	0.386207000	0.002089000
16	3.200307000	1.229794000	-0.015680000
8	3.357786000	1.909018000	1.249724000
8	3.339930000	1.885634000	-1.295450000
6	4.497686000	-0.133099000	-0.012610000
9	4.340500000	-0.906361000	-1.089751000
9	4.358251000	-0.884936000	1.081925000
9	5.708887000	0.404501000	-0.027825000
6	1.010103000	0.163378000	1.228641000
1	1.641965000	0.375810000	2.087461000
6	0.631979000	-1.320158000	1.284904000
7	-0.016389000	-1.757146000	0.033755000
16	-0.766626000	-3.240984000	0.050119000
8	-0.477080000	-3.938623000	-1.182282000
8	-0.595903000	-3.838634000	1.354911000
6	-2.585071000	-2.770993000	-0.057867000
9	-2.810892000	-2.097914000	-1.184773000
9	-3.313390000	-3.881894000	-0.053353000
9	-2.920694000	-2.015574000	0.984201000
6	0.621016000	-1.346528000	-1.231495000
1	-0.066104000	-1.543830000	-2.049956000
1	1.531703000	-1.923026000	-1.411581000
1	-0.049027000	-1.499516000	2.112173000
1	1.543399000	-1.894418000	1.469696000
6	-0.154158000	1.161580000	1.261858000
1	-0.819639000	0.941129000	2.091777000
1	0.266832000	2.160808000	1.405866000
1	1.613620000	0.337487000	-2.080260000

1-c-out-2-c-in

7	-0.204526000	1.163695000	0.020295000
16	-1.232422000	2.450397000	0.024019000
8	-1.898387000	2.497810000	1.303377000
8	-1.930147000	2.480344000	-1.238847000
6	-0.114069000	3.971374000	0.000110000
9	-0.848836000	5.073887000	0.005026000
9	0.682253000	3.963957000	1.074457000
9	0.651423000	3.953162000	-1.096240000
6	0.432410000	0.749235000	-1.239528000
1	-0.263237000	0.940294000	-2.051828000
1	1.345878000	1.322980000	-1.419384000
6	0.809236000	-0.736588000	-1.198641000
7	1.609856000	-0.965442000	0.015379000
16	3.036679000	-1.771719000	0.006968000
8	3.184399000	-2.455731000	-1.256561000
8	3.222135000	-2.410552000	1.288993000
6	4.299375000	-0.377217000	-0.039631000
9	4.111214000	0.363722000	-1.135862000
9	5.524336000	-0.881674000	-0.053689000
9	4.152007000	0.402293000	1.035276000
6	0.827868000	-0.724074000	1.239168000
1	1.459947000	-0.927600000	2.100021000
6	-0.339994000	-1.716627000	1.289428000
7	-1.120965000	-1.687814000	0.038148000
16	-2.600080000	-2.447362000	0.049502000
8	-2.770570000	-3.186646000	-1.180708000
8	-2.822225000	-3.023764000	1.355982000
6	-3.793632000	-0.998609000	-0.074092000
9	-3.640894000	-0.192195000	0.972925000
9	-3.561853000	-0.317207000	-1.195397000
9	-5.035544000	-1.469647000	-0.092663000
6	-0.355948000	-1.732777000	-1.222300000
1	-1.024700000	-1.504578000	-2.047900000
1	0.057890000	-2.730230000	-1.390700000
1	-0.998956000	-1.477952000	2.119513000
1	0.075399000	-2.712425000	1.465307000
6	0.455456000	0.762370000	1.272324000
1	-0.224543000	0.963455000	2.095317000
1	1.373051000	1.336403000	1.428729000
1	1.429072000	-0.945374000	-2.067088000

1-b-out-2-c-out

7	2.099205000	0.044663000	-0.000337000
16	3.335076000	-1.039016000	-0.000183000
8	3.338356000	-1.726436000	-1.270343000
8	3.337639000	-1.726950000	1.269702000
6	4.894017000	0.020200000	0.000510000
9	4.904170000	0.797854000	1.086242000
9	5.969214000	-0.753984000	0.000561000
9	4.904703000	0.798466000	-1.084771000
6	1.633560000	0.667026000	1.247388000
1	1.969214000	0.062857000	2.085732000
1	2.044097000	1.674670000	1.349498000
6	0.102149000	0.745367000	1.221696000
7	-0.306000000	1.446006000	-0.000496000
16	-0.880734000	2.983730000	-0.000590000
8	-0.566221000	3.591991000	-1.273088000
8	-0.564664000	3.592844000	1.271116000
6	-2.749475000	2.761142000	0.000729000
9	-3.333540000	3.952759000	0.000741000
9	-3.117418000	2.084577000	1.087485000
9	-3.118929000	2.083837000	-1.085015000
6	0.102154000	0.745133000	-1.222542000
1	-0.223588000	1.329044000	-2.077776000
6	-0.564007000	-0.666960000	-1.249710000
7	-1.284191000	-0.909472000	-0.000282000
16	-2.771360000	-1.607371000	-0.000181000
8	-3.407249000	-1.348707000	1.269734000
8	-3.407123000	-1.349261000	-1.270272000
6	-2.399845000	-3.454028000	0.000254000
9	-1.687809000	-3.768036000	1.085910000
9	-3.531752000	-4.144965000	0.000272000
9	-1.687564000	-3.768493000	-1.085110000
6	-0.563947000	-0.666761000	1.249074000
1	0.199604000	-1.432251000	1.402471000
1	-1.276911000	-0.723476000	2.064560000
1	0.199487000	-1.432518000	-1.403081000
1	-1.277036000	-0.723733000	-2.065135000
6	1.633568000	0.666769000	-1.248187000
1	1.969236000	0.062428000	-2.086397000
1	2.044110000	1.674393000	-1.350507000
1	-0.223651000	1.329401000	2.076821000

1-c-out-2-b-out

7	-1.703372000	0.731513000	-0.000098000
16	-2.525143000	2.155851000	-0.000490000
8	-2.307578000	2.806296000	-1.271053000
8	-2.306688000	2.807539000	1.269285000
6	-4.341654000	1.653058000	0.000362000
9	-4.601016000	0.919292000	1.086274000
9	-5.112569000	2.730292000	-0.000049000
9	-4.601612000	0.918014000	-1.084527000
6	-1.479198000	-0.013581000	1.246380000
1	-1.579225000	0.670376000	2.084480000
1	-2.215548000	-0.814296000	1.351437000
6	-0.072255000	-0.618864000	1.220085000
7	0.039915000	-1.427156000	0.000554000
16	0.765606000	-2.907385000	0.000867000
8	1.407597000	-3.122744000	1.275362000
8	1.409945000	-3.122419000	-1.272494000
6	-0.727172000	-4.052941000	-0.000763000
9	-1.473759000	-3.827297000	1.084486000
9	-0.322145000	-5.314849000	-0.000961000
9	-1.471939000	-3.826385000	-1.087173000
6	-0.072095000	-0.619502000	-1.219423000
1	0.039476000	-1.276846000	-2.076110000
6	1.047937000	0.469192000	-1.248403000
7	1.809191000	0.428407000	0.000176000
16	3.438517000	0.663966000	0.000308000
8	3.967414000	0.232418000	1.271212000
8	3.967784000	0.231486000	-1.270136000
6	3.623971000	2.539474000	-0.000433000
9	3.035016000	3.049956000	-1.086129000
9	3.034756000	3.050905000	1.084668000
9	4.908416000	2.869287000	-0.000428000
6	1.047582000	0.470075000	1.248475000
1	0.604054000	1.457695000	1.396253000
1	1.732175000	0.270779000	2.066021000
1	0.604676000	1.456787000	-1.397181000
1	1.732747000	0.269011000	-2.065548000
6	-1.479013000	-0.014207000	-1.246145000
1	-1.579034000	0.669295000	-2.084610000
1	-2.215272000	-0.815058000	-1.350855000
1	0.039404000	-1.275797000	2.077070000

Cartesian coordinates of 3,7,9-tri(trifluoromethylsulfonil)-3,7,9-triazabicyclo[3.3.1]nonane calculated at B3LYP/aug-cc-pVTZ level

1-c-out-2-c-out			
7	0.192083000	1.794562000	0.000000000
6	0.750702000	1.265470000	1.253645000
6	0.809534000	-0.267396000	1.220979000
6	0.809534000	-0.267396000	-1.220979000
6	-0.543760000	-0.987151000	1.254760000
7	-1.283930000	-0.777521000	0.000000000
6	-0.543760000	-0.987151000	-1.254760000
7	1.522460000	-0.685668000	0.000000000
16	3.048233000	-1.239802000	0.000000000
16	-0.672244000	3.177435000	0.000000000
6	2.834348000	-3.099669000	0.000000000
6	0.616508000	4.540095000	0.000000000
8	3.654080000	-0.938780000	-1.261987000
8	3.654080000	-0.938780000	1.261987000
8	-1.336015000	3.300454000	-1.260908000
8	-1.336015000	3.300454000	1.260908000
9	2.151325000	-3.479928000	-1.085642000
9	4.021312000	-3.692098000	0.000000000
9	2.151325000	-3.479928000	1.085642000
9	0.029035000	5.730092000	0.000000000
9	1.392826000	4.432149000	-1.085489000
9	1.392826000	4.432149000	1.085489000
16	-2.915395000	-0.787177000	0.000000000
8	-3.374671000	-0.292921000	-1.260810000
8	-3.374671000	-0.292921000	1.260810000
6	-3.376725000	-2.606100000	0.000000000
9	-2.869355000	-3.204817000	1.085395000
9	-2.869355000	-3.204817000	-1.085395000
9	-4.695828000	-2.747926000	0.000000000
6	0.750702000	1.265470000	-1.253645000
1	-0.357958000	-2.052402000	-1.416400000
1	-1.147843000	-0.611275000	-2.075678000
1	-0.357958000	-2.052402000	1.416400000
1	-1.147843000	-0.611275000	2.075678000
1	1.383504000	-0.590914000	-2.085735000
1	1.383504000	-0.590914000	2.085735000
1	0.126684000	1.601887000	2.076809000
1	1.766825000	1.638764000	1.408521000

1	0.126684000	1.601887000	-2.076809000
1	1.766825000	1.638764000	-1.408521000

1-c-in-2-c-out

7	0.997960000	-1.094098000	0.019565000
16	2.600131000	-1.401640000	0.035887000
8	3.122924000	-1.021127000	1.312036000
8	3.159023000	-0.969070000	-1.207994000
6	2.736933000	-3.273352000	-0.000293000
9	2.115342000	-3.795734000	1.063890000
9	2.153911000	-3.751492000	-1.106184000
9	4.012170000	-3.639993000	0.014783000
6	0.245944000	-1.165066000	-1.244220000
1	0.911571000	-0.885597000	-2.055879000
1	-0.113530000	-2.182584000	-1.421116000
6	-0.974822000	-0.236535000	-1.210794000
7	-1.764538000	-0.539126000	-0.005115000
16	-3.159048000	-1.370572000	-0.030652000
8	-3.319145000	-2.049001000	1.220121000
8	-3.294830000	-2.012278000	-1.303485000
6	-4.465213000	-0.029205000	-0.023638000
9	-4.317047000	0.755638000	-1.096206000
9	-4.339288000	0.722667000	1.075057000
9	-5.673920000	-0.575854000	-0.044334000
6	-1.000338000	-0.269047000	1.224809000
1	-1.622175000	-0.518433000	2.081102000
6	-0.703962000	1.232246000	1.290162000
7	-0.087297000	1.723389000	0.040276000
16	0.561322000	3.236371000	0.063472000
8	0.221222000	3.919432000	-1.149297000
8	0.368771000	3.803994000	1.364708000
6	2.399483000	2.906509000	-0.066133000
9	2.667882000	2.266330000	-1.204873000
9	3.046017000	4.068912000	-0.053904000
9	2.807709000	2.164229000	0.961061000
6	-0.684775000	1.267018000	-1.231259000
1	-0.000799000	1.498895000	-2.043269000
1	-1.622884000	1.792324000	-1.423791000
1	-0.031096000	1.441402000	2.117043000
1	-1.643911000	1.754498000	1.483383000
6	0.215085000	-1.203075000	1.261290000
1	0.861088000	-0.951308000	2.097382000
1	-0.151577000	-2.224332000	1.397129000
1	-1.576729000	-0.467425000	-2.086246000

1-c-out-2-c-in

7	-1.162262000	-0.172021000	0.000000000
16	-2.468507000	-1.151997000	0.000000000
8	-2.532059000	-1.825199000	1.260483000
8	-2.532059000	-1.825199000	-1.260483000
6	-3.948201000	0.003666000	0.000000000
9	-5.074624000	-0.697303000	0.000000000
9	-3.916737000	0.786882000	1.085448000
9	-3.916737000	0.786882000	-1.085448000
6	-0.727666000	0.463256000	-1.255394000
1	-0.942916000	-0.217196000	-2.074274000
1	-1.273621000	1.395170000	-1.424595000
6	0.769005000	0.791316000	-1.217981000
7	1.038131000	1.574523000	0.000000000
16	1.828342000	2.992920000	0.000000000
8	2.482167000	3.161625000	-1.262323000
8	2.482167000	3.161625000	1.262323000
6	0.448169000	4.258227000	0.000000000
9	-0.316994000	4.097924000	-1.085837000
9	0.956350000	5.483410000	0.000000000
9	-0.316994000	4.097924000	1.085837000
6	0.769005000	0.791316000	1.217981000
1	0.992732000	1.409595000	2.083854000
6	1.727949000	-0.403866000	1.258937000
7	1.689788000	-1.174715000	0.000000000
16	2.386043000	-2.665262000	0.000000000
8	3.030016000	-2.883873000	-1.260878000
8	3.030016000	-2.883873000	1.260878000
6	0.909134000	-3.814977000	0.000000000
9	0.166047000	-3.604982000	1.086617000
9	0.166047000	-3.604982000	-1.086617000
9	1.340863000	-5.072990000	0.000000000
6	1.727949000	-0.403866000	-1.258937000
1	1.466138000	-1.063092000	-2.081924000
1	2.734323000	-0.019875000	-1.442673000
1	1.466138000	-1.063092000	2.081924000
1	2.734323000	-0.019875000	1.442673000
6	-0.727666000	0.463256000	1.255394000
1	-0.942916000	-0.217196000	2.074274000
1	-1.273621000	1.395170000	1.424595000
1	0.992732000	1.409595000	-2.083854000

1-b-out-2-c-out

7	0.075005000	2.089413000	0.000000000
16	-0.995167000	3.318329000	0.000000000
8	-1.675939000	3.329466000	1.259266000
8	-1.675939000	3.329466000	-1.259266000
6	0.062098000	4.866166000	0.000000000
9	0.843085000	4.878724000	-1.085498000
9	-0.707381000	5.946866000	0.000000000
9	0.843085000	4.878724000	1.085498000
6	0.693908000	1.612823000	-1.246903000
1	0.093688000	1.950300000	-2.087063000
1	1.703985000	2.016741000	-1.348649000
6	0.762665000	0.081068000	-1.220746000
7	1.466259000	-0.330509000	0.000000000
16	2.981269000	-0.924688000	0.000000000
8	3.590234000	-0.625192000	1.261333000
8	3.590234000	-0.625192000	-1.261333000
6	2.741315000	-2.780992000	0.000000000
9	3.926660000	-3.381592000	0.000000000
9	2.059765000	-3.147789000	-1.086161000
9	2.059765000	-3.147789000	1.086161000
6	0.762665000	0.081068000	1.220746000
1	1.342861000	-0.246357000	2.077769000
6	-0.651643000	-0.577583000	1.248822000
7	-0.899448000	-1.301474000	0.000000000
16	-1.666208000	-2.739696000	0.000000000
8	-1.444838000	-3.381778000	-1.259309000
8	-1.444838000	-3.381778000	1.259309000
6	-3.484646000	-2.286020000	0.000000000
9	-3.770346000	-1.558409000	-1.085476000
9	-4.231432000	-3.383785000	0.000000000
9	-3.770346000	-1.558409000	1.085476000
6	-0.651643000	-0.577583000	-1.248822000
1	-1.413517000	0.189696000	-1.400785000
1	-0.711928000	-1.287676000	-2.066360000
1	-1.413517000	0.189696000	1.400785000
1	-0.711928000	-1.287676000	2.066360000
6	0.693908000	1.612823000	1.246903000
1	0.093688000	1.950300000	2.087063000
1	1.703985000	2.016741000	1.348649000
1	1.342861000	-0.246357000	-2.077769000

1-c-out-2-b-out

7	-0.759333000	1.679011000	0.000000000
16	-2.215031000	2.410792000	0.000000000
8	-2.849110000	2.164447000	1.259357000
8	-2.849110000	2.164447000	-1.259357000
6	-1.820226000	4.242911000	0.000000000
9	-1.101001000	4.549096000	-1.085473000
9	-2.939662000	4.954409000	0.000000000
9	-1.101001000	4.549096000	1.085473000
6	-0.006523000	1.467546000	-1.245304000
1	-0.689491000	1.550730000	-2.085782000
1	0.775066000	2.223988000	-1.348327000
6	0.631797000	0.075226000	-1.219153000
7	1.442433000	-0.029185000	0.000000000
16	2.954199000	-0.641368000	0.000000000
8	3.210841000	-1.261928000	-1.263127000
8	3.210841000	-1.261928000	1.263127000
6	4.009181000	0.905099000	0.000000000
9	3.743685000	1.641430000	-1.085713000
9	5.294334000	0.575510000	0.000000000
9	3.743685000	1.641430000	1.085713000
6	0.631797000	0.075226000	1.219153000
1	1.290217000	-0.020241000	2.076933000
6	-0.430884000	-1.069145000	1.248452000
7	-0.377788000	-1.833231000	0.000000000
16	-0.567016000	-3.453920000	0.000000000
8	-0.128582000	-3.969669000	-1.259898000
8	-0.128582000	-3.969669000	1.259898000
6	-2.426196000	-3.692696000	0.000000000
9	-2.958312000	-3.118883000	1.085411000
9	-2.958312000	-3.118883000	-1.085411000
9	-2.722887000	-4.986860000	0.000000000
6	-0.430884000	-1.069145000	-1.248452000
1	-1.427990000	-0.648409000	-1.396882000
1	-0.215795000	-1.747241000	-2.067139000
1	-1.427990000	-0.648409000	1.396882000
1	-0.215795000	-1.747241000	2.067139000
6	-0.006523000	1.467546000	1.245304000
1	-0.689491000	1.550730000	2.085782000
1	0.775066000	2.223988000	1.348327000
1	1.290217000	-0.020241000	-2.076933000

Cartesian coordinates of 3,7,9-tri(trifluoromethylsulfonil)-3,7,9-triazabicyclo[3.3.1]nonane calculated at M06-2X/cc-pVTZ level

1-c-out-2-c-out			
7	0.229069000	-1.480968000	0.000125000
16	-0.595471000	-2.880877000	0.000123000
8	-1.253135000	-3.029485000	-1.264433000
8	-1.252908000	-3.029613000	1.264781000
6	0.755367000	-4.143809000	-0.000069000
9	1.513327000	-3.971662000	-1.078625000
9	1.513463000	-3.971840000	1.078404000
9	0.260776000	-5.363645000	-0.000145000
6	0.764503000	-0.933422000	1.246207000
1	0.180967000	-1.327987000	2.074920000
1	1.811779000	-1.227022000	1.368435000
6	0.690052000	0.590936000	1.224112000
7	1.367387000	1.046860000	0.000062000
16	2.305189000	2.374611000	0.000033000
8	2.185399000	3.045458000	1.264036000
8	2.185292000	3.045520000	-1.263926000
6	3.987152000	1.611883000	-0.000049000
9	4.909305000	2.552443000	-0.000079000
9	4.125876000	0.853124000	-1.079158000
9	4.125971000	0.853130000	1.079051000
6	0.690186000	0.590828000	-1.224020000
1	1.233847000	0.975947000	-2.084135000
6	-0.724613000	1.169724000	-1.248992000
7	-1.417656000	0.851264000	-0.000088000
16	-3.000567000	0.490517000	-0.000131000
8	-3.342191000	-0.093270000	-1.264356000
8	-3.342182000	-0.093567000	1.263958000
6	-3.792253000	2.160742000	0.000044000
9	-5.104768000	2.058922000	-0.000037000
9	-3.398120000	2.828999000	1.078593000
9	-3.398000000	2.829236000	-1.078314000
6	-0.724760000	1.169812000	1.248872000
1	-1.294748000	0.751825000	2.075606000
1	-0.648141000	2.254508000	1.375469000
1	-1.294518000	0.751707000	-2.075765000
1	-0.647948000	2.254411000	-1.375642000
6	0.764610000	-0.933533000	-1.245966000
1	0.181117000	-1.328147000	-2.074688000

1	1.811881000	-1.227189000	-1.368095000
1	1.233609000	0.976153000	2.084248000

1-c-in-2-c-out

7	-0.967048000	-0.897367000	0.000000000
16	-1.576337000	-2.412862000	0.000000000
8	-1.285253000	-3.024150000	1.264064000
8	-1.285253000	-3.024150000	-1.264064000
6	-3.414714000	-2.175047000	0.000000000
9	-3.777346000	-1.490284000	1.078420000
9	-3.777346000	-1.490284000	-1.078420000
9	-4.018461000	-3.345430000	0.000000000
6	-1.018761000	-0.125775000	-1.249501000
1	-0.815106000	-0.803319000	-2.075636000
1	-2.007968000	0.322245000	-1.387251000
6	0.000204000	1.011721000	-1.219802000
7	-0.211384000	1.800410000	0.000000000
16	-1.074479000	3.174943000	0.000000000
8	-1.738982000	3.318201000	1.264391000
8	-1.738982000	3.318201000	-1.264391000
6	0.273231000	4.438112000	0.000000000
9	1.027183000	4.272338000	-1.079078000
9	1.027183000	4.272338000	1.079078000
9	-0.239070000	5.650856000	0.000000000
6	0.000204000	1.011721000	1.219802000
1	-0.192474000	1.648805000	2.080655000
6	1.467584000	0.598802000	1.253847000
7	1.849780000	-0.057211000	0.000000000
16	3.170727000	-1.018290000	0.000000000
8	3.841870000	-0.900994000	-1.263874000
8	3.841870000	-0.900994000	1.263874000
6	2.391637000	-2.690661000	0.000000000
9	1.636929000	-2.820507000	-1.080090000
9	3.328799000	-3.618711000	0.000000000
9	1.636929000	-2.820507000	1.080090000
6	1.467584000	0.598802000	-1.253847000
1	1.646895000	-0.088714000	-2.077123000
1	2.072344000	1.495946000	-1.413980000
1	1.646895000	-0.088714000	2.077123000
1	2.072344000	1.495946000	1.413980000
6	-1.018761000	-0.125775000	1.249501000
1	-0.815106000	-0.803319000	2.075636000
1	-2.007968000	0.322245000	1.387251000
1	-0.192474000	1.648805000	-2.080655000

1-c-out-2-c-in

7	-1.077716000	-0.429231000	0.000000000
16	-2.274418000	-1.539753000	0.000000000
8	-2.273145000	-2.215881000	1.264444000
8	-2.273145000	-2.215881000	-1.264444000
6	-3.834129000	-0.537600000	0.000000000
9	-4.882543000	-1.333085000	0.000000000
9	-3.867142000	0.237905000	1.078247000
9	-3.867142000	0.237905000	-1.078247000
6	-0.790165000	0.288826000	-1.248581000
1	-0.892022000	-0.412306000	-2.073660000
1	-1.494023000	1.114964000	-1.387428000
6	0.617150000	0.878251000	-1.219382000
7	0.729225000	1.688899000	0.000000000
16	1.482018000	3.129174000	0.000000000
8	2.131939000	3.327011000	-1.264531000
8	2.131939000	3.327011000	1.264531000
6	0.034490000	4.276329000	0.000000000
9	-0.703964000	4.050515000	-1.078974000
9	0.445494000	5.526966000	0.000000000
9	-0.703964000	4.050515000	1.078974000
6	0.617150000	0.878251000	1.219382000
1	0.720574000	1.535236000	2.080667000
6	1.765491000	-0.125364000	1.255646000
7	1.828287000	-0.881837000	0.000000000
16	2.624523000	-2.308642000	0.000000000
8	3.281471000	-2.487340000	-1.263763000
8	3.281471000	-2.487340000	1.263763000
6	1.209747000	-3.491537000	0.000000000
9	0.471115000	-3.285430000	1.080061000
9	0.471115000	-3.285430000	-1.080061000
9	1.657659000	-4.731504000	0.000000000
6	1.765491000	-0.125364000	-1.255646000
1	1.633814000	-0.824467000	-2.078234000
1	2.695255000	0.428156000	-1.417143000
1	1.633814000	-0.824467000	2.078234000
1	2.695255000	0.428156000	1.417143000
6	-0.790165000	0.288826000	1.248581000
1	-0.892022000	-0.412306000	2.073660000
1	-1.494023000	1.114964000	1.387428000
1	0.720574000	1.535236000	-2.080667000

1-b-out-2-c-out

7	0.403563000	2.092647000	0.000000000
16	-0.813276000	3.163759000	0.000000000
8	-1.493942000	3.092908000	1.262437000
8	-1.493942000	3.092908000	-1.262437000
6	0.063688000	4.790176000	0.000000000
9	0.831147000	4.870673000	-1.078594000
9	-0.805828000	5.778798000	0.000000000
9	0.831147000	4.870673000	1.078594000
6	1.007330000	1.610287000	-1.243677000
1	0.434337000	1.990177000	-2.085859000
1	2.038138000	1.964760000	-1.313631000
6	0.991608000	0.082906000	-1.224143000
7	1.670190000	-0.338472000	0.000000000
16	2.951411000	-1.334290000	0.000000000
8	3.618965000	-1.210646000	1.264252000
8	3.618965000	-1.210646000	-1.264252000
6	2.189632000	-3.016947000	0.000000000
9	3.129927000	-3.940178000	0.000000000
9	1.434488000	-3.149851000	-1.080655000
9	1.434488000	-3.149851000	1.080655000
6	0.991608000	0.082906000	1.224143000
1	1.559958000	-0.289056000	2.072561000
6	-0.459112000	-0.473642000	1.244918000
7	-0.720889000	-1.192625000	0.000000000
16	-1.712240000	-2.485063000	0.000000000
8	-1.599761000	-3.156134000	-1.262304000
8	-1.599761000	-3.156134000	1.262304000
6	-3.397757000	-1.721703000	0.000000000
9	-3.534905000	-0.960374000	-1.078679000
9	-4.325150000	-2.657449000	0.000000000
9	-3.534905000	-0.960374000	1.078679000
6	-0.459112000	-0.473642000	-1.244918000
1	-1.171780000	0.346860000	-1.370985000
1	-0.582418000	-1.173640000	-2.066006000
1	-1.171780000	0.346860000	1.370985000
1	-0.582418000	-1.173640000	2.066006000
6	1.007330000	1.610287000	1.243677000
1	0.434337000	1.990177000	2.085859000
1	2.038138000	1.964760000	1.313631000
1	1.559958000	-0.289056000	-2.072561000

1-c-out-2-b-out			
7	1.957709000	0.406769000	-0.000074000
16	3.120001000	-0.722128000	-0.000175000
8	3.104559000	-1.405712000	-1.262879000
8	3.104801000	-1.405689000	1.262543000
6	4.668260000	0.285950000	-0.000375000
9	4.683444000	1.057844000	1.078191000
9	5.727107000	-0.495669000	-0.000770000
9	4.682896000	1.058180000	-1.078717000
6	1.407295000	0.956771000	1.237045000
1	1.840721000	0.430906000	2.083954000
1	1.652976000	2.019088000	1.304335000
6	-0.107031000	0.778028000	1.220056000
7	-0.606490000	1.426578000	0.000064000
16	-2.164370000	1.961308000	0.000160000
8	-2.775803000	1.670479000	1.265547000
8	-2.775789000	1.670892000	-1.265328000
6	-1.859215000	3.783380000	0.000483000
9	-1.165753000	4.118935000	1.079266000
9	-3.011194000	4.423179000	0.000682000
9	-1.165891000	4.119354000	-1.078252000
6	-0.107132000	0.778131000	-1.220022000
1	-0.533538000	1.293473000	-2.075994000
6	-0.520313000	-0.721032000	-1.240235000
7	-1.225314000	-1.030911000	-0.000015000
16	-2.489020000	-2.060008000	0.000005000
8	-3.159046000	-1.970324000	1.263615000
8	-3.159148000	-1.970251000	-1.263546000
6	-1.665685000	-3.715761000	-0.000067000
9	-0.899414000	-3.826808000	-1.078695000
9	-0.899203000	-3.826788000	1.078414000
9	-2.567352000	-4.676786000	0.000028000
6	-0.520200000	-0.721140000	1.240167000
1	0.364278000	-1.357605000	1.344427000
1	-1.196483000	-0.910567000	2.068499000
1	0.364152000	-1.357491000	-1.344633000
1	-1.196672000	-0.910377000	-2.068523000
6	1.407194000	0.956860000	-1.237113000
1	1.840551000	0.431055000	-2.084094000
1	1.652881000	2.019179000	-1.304344000
1	-0.533371000	1.293292000	2.076108000

Cartesian coordinates of 3,7,9-tri(trifluoromethylsulfonil)-3,7,9-triazabicyclo[3.3.1]nonane calculated at M06-2X/aug-cc-pVTZ level

1-c-out-2-c-out			
7	-0.710600000	1.480030000	0.000000000
6	-1.089860000	0.812469000	-1.249633000
6	-0.649578000	-0.650146000	-1.223320000
6	-0.649578000	-0.650146000	1.223320000
6	0.861346000	-0.871534000	-1.244523000
7	1.460254000	-0.391043000	0.000000000
6	0.861346000	-0.871534000	1.244523000
7	-1.177968000	-1.270922000	0.000000000
16	-2.471562000	-2.227079000	0.000000000
16	-0.353111000	3.056237000	0.000000000
6	-1.704252000	-3.898948000	0.000000000
6	-2.010676000	3.860103000	0.000000000
8	-3.136242000	-2.120409000	1.255180000
8	-3.136242000	-2.120409000	-1.255180000
8	0.224200000	3.396058000	1.254618000
8	0.224200000	3.396058000	-1.254618000
9	-0.940674000	-4.036528000	1.079047000
9	-2.635496000	-4.831941000	0.000000000
9	-0.940674000	-4.036528000	-1.079047000
9	-1.896776000	5.172948000	0.000000000
9	-2.688118000	3.475750000	1.078553000
9	-2.688118000	3.475750000	-1.078553000
16	2.839960000	0.440916000	0.000000000
8	2.986766000	1.094199000	1.255016000
8	2.986766000	1.094199000	-1.255016000
6	4.106736000	-0.894036000	0.000000000
9	3.941789000	-1.655077000	-1.078811000
9	3.941789000	-1.655077000	1.078811000
9	5.325700000	-0.392570000	0.000000000
6	-1.089860000	0.812469000	1.249633000
1	1.055140000	-1.941968000	1.360041000
1	1.311345000	-0.334799000	2.076050000
1	1.055140000	-1.941968000	-1.360041000
1	1.311345000	-0.334799000	-2.076050000
1	-1.088192000	-1.152606000	2.082843000
1	-1.088192000	-1.152606000	-2.082843000
1	-0.617283000	1.338763000	-2.075812000
1	-2.176260000	0.836642000	-1.379456000

1	-0.617283000	1.338763000	2.075812000
1	-2.176260000	0.836642000	1.379456000

1-c-in-2-c-out

7	-0.967869000	-0.941636000	0.000000000
16	-1.504251000	-2.472106000	0.000000000
8	-1.192215000	-3.066154000	1.254572000
8	-1.192215000	-3.066154000	-1.254572000
6	-3.343209000	-2.316287000	0.000000000
9	-3.738424000	-1.646751000	1.078570000
9	-3.738424000	-1.646751000	-1.078570000
9	-3.900215000	-3.511306000	0.000000000
6	-1.042716000	-0.169341000	-1.249013000
1	-0.815115000	-0.837126000	-2.076615000
1	-2.047313000	0.242663000	-1.385579000
6	-0.066524000	1.004605000	-1.218698000
7	-0.315604000	1.784043000	0.000000000
16	-1.160949000	3.154421000	0.000000000
8	-1.817743000	3.301706000	1.255091000
8	-1.817743000	3.301706000	-1.255091000
6	0.176988000	4.416968000	0.000000000
9	0.935018000	4.255594000	-1.079203000
9	0.935018000	4.255594000	1.079203000
9	-0.335485000	5.631101000	0.000000000
6	-0.066524000	1.004605000	1.218698000
1	-0.283712000	1.632824000	2.080173000
6	1.415019000	0.647526000	1.255280000
7	1.834336000	0.014587000	0.000000000
16	3.184993000	-0.883001000	0.000000000
8	3.844889000	-0.740450000	-1.254488000
8	3.844889000	-0.740450000	1.254488000
6	2.490182000	-2.583917000	0.000000000
9	1.740213000	-2.751579000	-1.080330000
9	3.467396000	-3.471569000	0.000000000
9	1.740213000	-2.751579000	1.080330000
6	1.415019000	0.647526000	-1.255280000
1	1.614751000	-0.037610000	-2.075801000
1	1.985125000	1.565009000	-1.424527000
1	1.614751000	-0.037610000	2.075801000
1	1.985125000	1.565009000	1.424527000
6	-1.042716000	-0.169341000	1.249013000
1	-0.815115000	-0.837126000	2.076615000
1	-2.047313000	0.242663000	1.385579000
1	-0.283712000	1.632824000	-2.080173000

1-c-out-2-c-in			
7	0.332425000	1.053490000	0.057117000
16	1.344022000	2.320273000	0.061387000
8	2.136161000	2.284108000	-1.119399000
8	1.882121000	2.468017000	1.370194000
6	0.241104000	3.783795000	-0.161711000
9	0.939428000	4.899006000	-0.093775000
9	-0.366975000	3.715914000	-1.342362000
9	-0.690152000	3.788621000	0.789626000
6	-0.420675000	0.751107000	1.281911000
1	0.221704000	0.953915000	2.135034000
1	-1.313234000	1.380164000	1.346766000
6	-0.868654000	-0.705675000	1.284042000
7	-1.620129000	-0.943822000	0.045663000
16	-3.056312000	-1.670006000	0.020840000
8	-3.298927000	-2.275881000	1.286532000
8	-3.219349000	-2.345199000	-1.222262000
6	-4.198492000	-0.230305000	-0.055500000
9	-4.010736000	0.536960000	1.013696000
9	-5.451908000	-0.634769000	-0.090133000
9	-3.934383000	0.487158000	-1.142507000
6	-0.785336000	-0.791951000	-1.151715000
1	-1.403979000	-0.972307000	-2.028519000
6	0.300396000	-1.861699000	-1.115260000
7	1.034997000	-1.798405000	0.153330000
16	2.535263000	-2.411058000	0.198794000
8	2.845399000	-2.773334000	1.541126000
8	2.738724000	-3.277928000	-0.913702000
6	3.547522000	-0.922294000	-0.174890000
9	3.184624000	-0.442403000	-1.359277000
9	3.349517000	0.004383000	0.749354000
9	4.826528000	-1.246506000	-0.206621000
6	0.244404000	-1.746150000	1.389424000
1	0.907576000	-1.511566000	2.218287000
1	-0.221054000	-2.717646000	1.579389000
1	1.000249000	-1.721583000	-1.935507000
1	-0.176939000	-2.839017000	-1.230824000
6	-0.298066000	0.655207000	-1.209556000
1	0.433404000	0.778018000	-2.005591000
1	-1.165327000	1.289069000	-1.417936000
1	-1.540745000	-0.851002000	2.127421000

1-b-out-2-c-out

7	0.493813000	2.070838000	0.000000000
16	-0.640656000	3.213955000	0.000000000
8	-1.318748000	3.193072000	1.252783000
8	-1.318748000	3.193072000	-1.252783000
6	0.340341000	4.770466000	0.000000000
9	1.114512000	4.799032000	-1.078647000
9	-0.454092000	5.821533000	0.000000000
9	1.114512000	4.799032000	1.078647000
6	1.072472000	1.551966000	-1.242102000
1	0.527885000	1.966227000	-2.086672000
1	2.123190000	1.843410000	-1.307050000
6	0.965127000	0.028350000	-1.223254000
7	1.617435000	-0.437430000	0.000000000
16	2.867118000	-1.454073000	0.000000000
8	3.532730000	-1.352441000	1.254822000
8	3.532730000	-1.352441000	-1.254822000
6	2.068413000	-3.110461000	0.000000000
9	2.984784000	-4.059521000	0.000000000
9	1.307950000	-3.228072000	-1.080395000
9	1.307950000	-3.228072000	1.080395000
6	0.965127000	0.028350000	1.223254000
1	1.508296000	-0.376389000	2.072973000
6	-0.515391000	-0.442731000	1.243044000
7	-0.820772000	-1.148381000	0.000000000
16	-1.844188000	-2.399598000	0.000000000
8	-1.758667000	-3.068199000	-1.253105000
8	-1.758667000	-3.068199000	1.253105000
6	-3.497240000	-1.587468000	0.000000000
9	-3.612973000	-0.819968000	-1.078810000
9	-4.457295000	-2.491715000	0.000000000
9	-3.612973000	-0.819968000	1.078810000
6	-0.515391000	-0.442731000	-1.243044000
1	-1.179305000	0.418722000	-1.362620000
1	-0.680928000	-1.129678000	-2.067398000
1	-1.179305000	0.418722000	1.362620000
1	-0.680928000	-1.129678000	2.067398000
6	1.072472000	1.551966000	1.242102000
1	0.527885000	1.966227000	2.086672000
1	2.123190000	1.843410000	1.307050000
1	1.508296000	-0.376389000	-2.072973000

1-c-out-2-b-out

7	1.932299000	0.111757000	0.000015000
16	3.002787000	-1.090884000	0.000020000
8	2.940749000	-1.765035000	-1.253192000
8	2.940728000	-1.765038000	1.253228000
6	4.618284000	-0.210342000	0.000034000
9	4.698007000	0.560661000	1.078762000
9	5.615543000	-1.071878000	0.000048000
9	4.698030000	0.560651000	-1.078699000
6	1.452537000	0.727593000	1.237192000
1	1.815625000	0.151173000	2.084211000
1	1.827466000	1.751457000	1.306428000
6	-0.072184000	0.738266000	1.219680000
7	-0.492339000	1.436618000	0.000004000
16	-1.881610000	2.283696000	0.000007000
8	-2.533977000	2.132425000	1.255988000
8	-2.533984000	2.132444000	-1.255973000
6	-1.204593000	3.994733000	0.000015000
9	-0.451016000	4.175618000	1.079061000
9	-2.187757000	4.873577000	0.000172000
9	-0.451268000	4.175729000	-1.079188000
6	-0.072167000	0.738283000	-1.219677000
1	-0.430382000	1.303073000	-2.075638000
6	-0.668877000	-0.697077000	-1.240840000
7	-1.404889000	-0.927340000	-0.000019000
16	-2.768844000	-1.797882000	-0.000028000
8	-3.420412000	-1.636680000	1.253573000
8	-3.420421000	-1.636657000	-1.253621000
6	-2.137301000	-3.528356000	-0.000045000
9	-1.384174000	-3.721717000	-1.078802000
9	-1.383993000	-3.721678000	1.078591000
9	-3.132641000	-4.393057000	0.000053000
6	-0.668887000	-0.697097000	1.240810000
1	0.128512000	-1.437883000	1.352194000
1	-1.364785000	-0.798144000	2.068109000
1	0.128518000	-1.437864000	-1.352235000
1	-1.364769000	-0.798102000	-2.068148000
6	1.452555000	0.727605000	-1.237165000
1	1.815653000	0.151191000	-2.084183000
1	1.827489000	1.751468000	-1.306387000
1	-0.430416000	1.303041000	2.075644000

Equation S1. Disagreement factor being minimized at least-squares procedure of the GED data.

$$R_f = 100\left[\frac{\sum_i^n \omega_i\left[s_i M_{\text{exp.}}(\mathrm{s_i}) - \mathrm{ks_i M_{theor.}(s_i)}\right]^2}{\sum_i^n \omega_i\left[s_i M_{\text{exp.}}(\mathrm{s_i})\right]^2}\right]^{1/2}$$

where: ω_i – weight function; k– scale coefficient (index of resolution), $k = s_i M_{exp.}(s_i)/s_i M_{theor.}(s_i)$, $s_i M_{exp.}(s_i)$, $s_i M_{theor.}(s_i)$ – experimental and theoretical molecular scattering intensities, respectively.

Equation S2. Molecular scattering intensities

$$sM(s) = \frac{I(s) - B}{B} \times s$$

where I(s) - molecular intensity, B - background function, $s = \frac{4\pi}{\lambda}\sin\frac{\theta}{2}$, λ – electron wavelength, θ – scattering angle

Table S1. Selected geometric parameters (Å and degrees) of 1 according to QC calculations.

Parameter	M06-2X/aug-cc-pVTZ					M06-2X/cc-pVTZ				
	I	II	III	IV	V	I	II	III	IV	V
rSN	1.609	1.610	1.610	1.611	1.627	1.626	1.623	1.623	1.623	1.647
rSC	1.840	1.840	1.839	1.839	1.840	1.847	1.847	1.847	1.847	1.847
rCF (ave)	1.326	1.325	1.325	1.325	1.325	1.324	1.323	1.324	1.323	1.323
$rNC_{\alpha}(f)$	1.470	1.468	1.468	1.462	1.467	1.471	1.469	1.468	1.462	1.469
$rNC_{\alpha}(n)$	1.470	1.468	1.468	1.462	1.467	1.471	1.468	1.468	1.462	1.469
$rC_{\alpha}C_{\beta}(1f)$	1.527	1.524	1.524	1.554	1.525	1.526	1.526	1.524	1.554	1.525
$rC_{\alpha}C_{\beta}(2f)$	1.528	1.527	1.527	1.528	1.555	1.529	1.526	1.527	1.528	1.555
$rC_{\alpha}C_{\beta}(1n)$	1.527	1.524	1.528	1.554	1.525	1.526	1.524	1.528	1.554	1.525
$rC_{\alpha}C_{\beta}(2n)$	1.528	1.527	1.525	1.528	1.555	1.529	1.529	1.525	1.528	1.555
$rC_{\beta}N(1f)$	1.462	1.467	1.469	1.462	1.463	1.463	1.466	1.469	1.461	1.461
$rC_{\beta}N(1n)$	1.462	1.467	1.470	1.462	1.463	1.463	1.466	1.469	1.461	1.461
$rC_{\beta}N(2f)$	1.467	1.470	1.468	1.465	1.461	1.463	1.468	1.467	1.464	1.460
$rC_{\beta}N(2n)$	1.467	1.470	1.467	1.465	1.461	1.463	1.469	1.466	1.464	1.460
rNS(1)	1.611	1.622	1.621	1.616	1.610	1.625	1.634	1.633	1.629	1.620
rNS(2)	1.616	1.622	1.621	1.611	1.618	1.624	1.632	1.633	1.621	1.630
rSC(1)	1.840	1.837	1.846	1.842	1.840	1.849	1.846	1.855	1.850	1.848
rSC(2)	1.842	1.846	1.839	1.840	1.842	1.848	1.853	1.846	1.848	1.849
rSO (ave)	1.423	1.424	1.424	1.424	1.423	1.434	1.435	1.435	1.435	1.435
N_tSC	101.8	101.7	101.6	103.4	99.8	100.8	101.0	100.9	103.5	99.4
$SN_tC_{\alpha}(f)$	122.7	122.8	122.9	123.2	119.5	121.2	122.0	122.4	122.9	117.7
$SN_tC_{\alpha}(n)$	122.7	122.8	122.8	123.2	119.5	121.2	121.9	121.9	122.9	117.7
$C_{\alpha}N_tC_{\alpha}$	112.6	112.2	112.4	113.5	112.5	112.6	112.4	112.5	113.8	112.3
$N_tC_{\alpha}C_{\beta}(1f)$	107.8	108.0	107.7	109.8	107.4	107.4	107.9	107.4	110.0	107.2
$N_tC_{\alpha}C_{\beta}(2f)$	108.4	108.4	108.7	107.3	109.9	108.8	108.5	109.0	107.1	110.2
$N_tC_{\alpha}C_{\beta}(1n)$	107.8	108.0	108.1	109.8	107.4	107.4	107.3	107.6	110.0	107.2
$N_tC_{\alpha}C_{\beta}(2n)$	108.4	108.4	108.2	107.3	109.9	108.8	108.9	108.6	107.1	110.2
$C_{\alpha}C_{\beta}N(1f)$	110.2	111.0	110.4	109.5	108.7	110.1	110.8	110.3	109.4	108.7
$C_{\alpha}C_{\beta}N(2f)$	110.3	110.8	111.0	108.4	109.1	110.0	110.1	110.7	108.3	108.8
$C_{\alpha}C_{\beta}N(1n)$	110.2	111.0	111.1	109.5	108.7	110.1	110.1	111.0	109.4	108.7
$C_{\alpha}C_{\beta}N(2n)$	110.3	110.8	110.3	108.4	109.1	110.0	110.8	110.0	108.3	108.8
$C_{\beta}NS(1f)$	121.4	118.5	118.6	120.4	122.2	120.6	118.6	118.3	120.0	122.2
$C_{\beta}NS(2f)$	120.1	118.6	119.2	122.0	120.6	120.6	118.4	119.0	121.8	120.6
$C_{\beta}NS(1n)$	121.4	118.5	118.8	120.4	122.2	120.7	117.7	118.4	120.0	122.2
$C_{\beta}NS(2n)$	120.1	118.6	118.1	122.0	120.6	120.7	118.7	118.0	121.8	120.6
NSC(1)	102.4	101.4	104.3	103.1	103.1	102.6	100.9	104.2	103.1	102.8

NSC(2)	103.1	104.5	101.4	103.0	102.5	102.5	104.3	100.8	103.0	102.7
flap(t1)	57.9	58.7	58.4	53.2	58.6	58.8	59.9	58.6	52.8	59.1
flap(t2)	57.2	57.5	57.6	58.5	53.6	56.2	56.1	57.5	58.8	53.0
flap(1)	47.1	42.9	45.4	48.9	51.7	47.2	46.0	44.9	59.0	51.7
flap(2)	46.4	45.3	44.2	52.1	50.1	46.9	45.4	45.9	52.0	50.9

Parameter	**B3LYP/aug-cc-pVTZ**					**B3LYP/cc-pVTZ**				
	**I**	**II**	**III**	**IV**	**V**	**I**	**II**	**III**	**IV**	**V**
rSN	1.623	1.624	1.624	1.627	1.631	1.639	1.640	1.639	1.642	1.649
rSC	1.872	1.872	1.872	1.872	1.872	1.882	1.882	1.882	1.882	1.882
rCF (ave)	1.335	1.333	1.334	1.333	1.334	1.333	1.332	1.332	1.332	1.333
rNC_{α}(f)	1.474	1.473	1.473	1.468	1.468	1.474	1.473	1.472	1.467	1.467
rNC_{α}(n)	1.474	1.473	1.473	1.468	1.468	1.474	1.472	1.472	1.467	1.467
$rC_{\alpha}C_{\beta}$(1f)	1.533	1.532	1.533	1.560	1.532	1.534	1.532	1.533	1.562	1.532
$rC_{\alpha}C_{\beta}$(2f)	1.534	1.533	1.533	1.534	1.562	1.535	1.534	1.533	1.534	1.562
$rC_{\alpha}C_{\beta}$(1n)	1.533	1.531	1.533	1.560	1.532	1.534	1.532	1.533	1.562	1.532
$rC_{\alpha}C_{\beta}$(2n)	1.534	1.534	1.533	1.534	1.562	1.535	1.535	1.533	1.534	1.562
$rC_{\beta}N$(1f)	1.472	1.478	1.473	1.465	1.470	1.470	1.475	1.471	1.462	1.469
$rC_{\beta}N$(1n)	1.472	1.477	1.473	1.465	1.470	1.470	1.475	1.471	1.462	1.469
$rC_{\beta}N$(2f)	1.471	1.472	1.477	1.471	1.465	1.469	1.471	1.475	1.470	1.463
$rC_{\beta}N$(2n)	1.471	1.472	1.477	1.471	1.465	1.469	1.471	1.475	1.470	1.463
rNS(1)	1.631	1.646	1.633	1.630	1.629	1.646	1.663	1.647	1.643	1.644
rNS(2)	1.631	1.632	1.645	1.630	1.632	1.644	1.647	1.663	1.644	1.646
rSC(1)	1.877	1.872	1.878	1.874	1.874	1.887	1.881	1.888	1.884	1.885
rSC(2)	1.876	1.877	1.645	1.874	1.874	1.885	1.888	1.881	1.885	1.885
rSO (ave)	1.431	1.432	1.432	1.432	1.431	1.443	1.445	1.444	1.444	1.443
$N_{t}SC$	103.4	103.4	103.4	104.0	102.3	102.7	102.6	102.7	103.7	101.4
$SN_{t}C_{\alpha}$(f)	123.5	123.5	123.6	123.3	122.6	122.9	122.9	123.1	123.0	121.9
$SN_{t}C_{\alpha}$(n)	123.5	123.6	123.6	123.3	122.6	122.9	122.9	123.2	123.0	121.9
$C_{\alpha}N_{t}C_{\alpha}$	111.8	111.6	111.6	112.5	112.3	112.1	111.7	111.8	112.9	112.5
$N_{t}C_{\alpha}C_{\beta}$(1f)	108.2	108.4	108.2	109.3	108.0	107.8	108.0	107.8	109.1	107.6
$N_{t}C_{\alpha}C_{\beta}$(2f)	108.6	108.6	108.8	108.4	109.8	108.8	108.9	109.0	108.4	110.1
$N_{t}C_{\alpha}C_{\beta}$(1n)	108.2	108.3	108.2	109.3	108.0	107.8	107.9	107.8	109.1	107.6
$N_{t}C_{\alpha}C_{\beta}$(2n)	108.6	108.7	108.8	108.4	109.8	108.8	108.9	109.0	108.4	110.1
$C_{\alpha}C_{\beta}N$(1f)	111.0	111.7	111.1	110.3	109.2	110.8	111.4	110.9	110.2	109.0
$C_{\alpha}C_{\beta}N$(2f)	110.8	110.9	111.6	109.1	110.0	110.6	110.7	111.2	108.9	109.7
$C_{\alpha}C_{\beta}N$(1n)	111.0	111.6	111.1	110.3	109.2	110.8	111.3	110.8	110.2	109.0
$C_{\alpha}C_{\beta}N$(2n)	110.8	111.0	111.6	109.1	110.0	110.6	110.8	111.3	108.9	109.7
$C_{\beta}NS$(1f)	120.1	117.3	119.7	121.0	121.5	119.8	117.0	119.5	121.1	121.0
$C_{\beta}NS$(2f)	120.4	120.0	117.5	121.4	120.9	120.3	119.5	117.0	121.1	120.7
$C_{\beta}NS$(1n)	120.1	117.1	119.7	121.0	121.5	119.8	116.9	119.4	121.1	121.0
$C_{\beta}NS$(2n)	120.4	120.1	117.5	121.4	120.9	120.3	119.6	117.1	121.1	120.7
NSC(1)	104.6	102.9	105.1	104.1	104.5	104.5	102.3	105.1	103.8	104.5
NSC(2)	104.6	105.0	102.9	104.6	104.0	104.4	105.1	102.2	104.6	103.9
flap(t1)	58.4	58.7	58.7	55.3	58.0	58.9	59.3	59.3	55.5	58.8
flap(t2)	57.4	57.6	57.7	57.1	54.5	56.8	57.0	57.0	56.9	53.7

flap(1)	43.8	41.0	43.3	46.1	49.7	44.2	42.4	43.7	46.1	50.0
flap(2)	44.4	44.1	41.6	49.8	46.9	44.8	44.4	43.2	50.3	47.3

Table S2. Disagreement factors R_f and refined conformers contribution X evaluated from least-squares refinement using the UNEX program. The $3\sigma_{LS}$ quantities were adopted as error limits

Method/basis [a]	R_f, %	X, mol. %				
		1-c-out-2-c-out	1-c-in-2-c-out	1-c-out-2-c-in	1-b-out-2-c-out	1-c-out-2-b-out
M062X/A	4.20	25(18)	48(57)	1(69)	17(20)	9(13)
M062X/B	4.20	42(27)	29(57)	11(57)	18(22)	0(6)
B3LYP/A	4.68	50(26)	0(84)	19(75)	26(33)	4(9)
B3LYP/B	4.36	32(23)	45(330)	4(51)	5[b]	14(230)

[a] A and B are cc-pVTZ and aug-cc-pVTZ basis sets, respectively.

[b] dependent parameter

Table S3. Dependence of agreement factor on the conformers ratio with a step of 2% in the region of minimal R-factors

Conformers contribution X, mol. %					R_f	Conformers contribution X, mol. %					R_f
I	II	III	IV	V		I	II	III	IV	V	
36	36	8	20	0	4.20	36	44	0	16	4	4.25
36	32	12	20	0	4.21	28	32	12	24	4	4.25
36	40	4	20	0	4.21	36	20	24	20	0	4.26
40	32	8	20	0	4.21	36	28	16	16	4	4.26
36	32	8	24	0	4.21	28	36	8	28	0	4.26
40	36	4	20	0	4.21	32	24	16	28	0	4.26
32	36	8	24	0	4.21	32	24	20	20	4	4.26
36	36	4	24	0	4.22	28	32	12	28	0	4.26
32	32	12	24	0	4.22	28	40	4	24	4	4.26
36	44	0	20	0	4.22	32	20	24	24	0	4.26
36	28	12	24	0	4.22	40	24	20	16	0	4.26
40	28	12	20	0	4.22	44	24	16	16	0	4.26
36	28	16	20	0	4.22	28	28	16	24	4	4.26
32	40	4	24	0	4.22	32	40	0	28	0	4.26
40	40	0	20	0	4.22	28	40	4	28	0	4.26
32	28	16	24	0	4.22	36	32	8	20	4	4.26
36	24	16	24	0	4.22	36	16	24	24	0	4.26
36	40	0	24	0	4.22	28	28	16	28	0	4.26
40	36	8	16	0	4.23	36	28	12	20	4	4.26
40	24	16	20	0	4.23	36	36	4	20	4	4.26
40	40	4	16	0	4.23	40	16	24	20	0	4.27
32	44	0	24	0	4.23	28	44	0	24	4	4.27
40	32	12	16	0	4.23	32	20	20	28	0	4.27
32	36	8	20	4	4.23	36	24	20	16	4	4.271
32	32	12	20	4	4.23	28	44	0	28	0	4.273
36	24	20	20	0	4.23	32	40	8	20	0	4.27
32	40	4	20	4	4.24	28	24	20	24	4	4.27
40	44	0	16	0	4.24	36	40	0	20	4	4.27
32	24	20	24	0	4.24	32	32	8	24	4	4.28
36	20	20	24	0	4.24	36	24	16	20	4	4.28
36	36	8	16	4	4.24	28	40	8	20	4	4.28
40	28	16	16	0	4.24	32	36	12	20	0	4.28
44	32	8	16	0	4.24	28	24	20	28	0	4.28
32	28	16	20	4	4.24	28	36	12	20	4	4.28
44	36	4	16	0	4.24	32	44	4	20	0	4.28

40	20	20	20	0	4.24	32	20	24	20	4	4.28
36	40	4	16	4	4.24	32	28	12	24	4	4.28
32	44	0	20	4	4.24	44	20	20	16	0	4.28
36	32	12	16	4	4.24	32	40	8	16	4	4.28
32	32	8	28	0	4.25	32	36	4	24	4	4.28
44	28	12	16	0	4.25	44	36	8	12	0	4.28
32	28	12	28	0	4.25	44	40	4	12	0	4.28
44	40	0	16	0	4.25	32	36	12	16	4	4.28
32	36	4	28	0	4.25	28	44	4	20	4	4.28
28	36	8	24	4	4.25	32	44	4	16	4	4.28
40	32	8	16	4	4.28	36	48	0	16	0	4.30
40	20	24	16	0	4.28	28	40	8	16	8	4.30
40	36	4	16	4	4.28	32	28	20	20	0	4.30
36	16	28	20	0	4.28	40	28	16	12	4	4.30
40	36	8	12	4	4.28	28	32	16	24	0	4.30
32	32	16	20	0	4.28	28	36	12	16	8	4.30
40	40	4	12	4	4.28	32	20	20	24	4	4.30
28	32	16	20	4	4.28	32	44	0	16	8	4.30
32	24	16	24	4	4.28	36	32	16	16	0	4.30
40	28	12	16	4	4.28	44	24	12	20	0	4.30
44	32	12	12	0	4.28	44	36	0	20	0	4.30
44	44	0	12	0	4.28	40	20	16	24	0	4.30
32	16	28	24	0	4.29	44	16	24	16	0	4.30
32	36	8	16	8	4.29	48	36	4	12	0	4.30
36	40	8	16	0	4.29	48	32	8	12	0	4.30
40	28	8	24	0	4.29	28	44	4	16	8	4.30
28	36	8	20	8	4.29	28	44	0	20	8	4.30
32	48	0	20	0	4.29	32	28	20	16	4	4.30
40	32	12	12	4	4.29	32	16	28	20	4	4.30
28	32	12	20	8	4.29	24	32	12	28	4	4.30
32	32	16	16	4	4.29	24	36	12	24	4	4.31
32	40	0	24	4	4.29	24	40	8	24	4	4.31
32	32	12	16	8	4.29	24	36	8	28	4	4.31
36	44	4	16	0	4.29	48	40	0	12	0	4.31
40	32	4	24	0	4.29	28	32	16	16	8	4.31
40	40	0	16	4	4.29	24	36	12	20	8	4.31
32	40	4	16	8	4.29	24	40	8	20	8	4.31
36	36	12	16	0	4.29	28	48	0	24	0	4.31
36	12	28	24	0	4.29	28	24	20	20	8	4.31
32	16	24	28	0	4.29	48	28	12	12	0	4.31
32	48	0	16	4	4.29	24	28	16	28	4	4.31

36	20	20	20	4	4.29	28	32	8	32	0	4.31
40	44	0	12	4	4.29	36	40	8	12	4	4.31
28	40	8	24	0	4.29	40	16	28	16	0	4.31
28	48	0	20	4	4.29	24	32	16	24	4	4.31
40	24	12	24	0	4.29	24	44	4	24	4	4.31
28	36	12	24	0	4.29	24	40	4	28	4	4.31
28	20	24	24	4	4.29	28	28	12	32	0	4.31
28	40	4	20	8	4.29	32	24	20	16	8	4.31
36	20	24	16	4	4.29	36	28	8	28	0	4.31
44	28	8	20	0	4.29	44	20	16	20	0	4.31
40	12	28	20	0	4.30	28	28	20	24	0	4.31
44	32	4	20	0	4.30	36	44	4	12	4	4.31
28	20	24	28	0	4.30	36	16	24	20	4	4.31
28	28	16	20	8	4.30	40	20	20	16	4	4.31
40	24	16	16	4	4.30	24	32	16	20	8	4.32
28	44	4	24	0	4.30	24	44	4	20	8	4.32
40	36	0	24	0	4.30	28	48	0	16	8	4.32
44	28	16	12	0	4.30	36	28	20	16	0	4.32
28	28	20	20	4	4.30	36	24	12	28	0	4.32
32	28	16	16	8	4.30	36	32	4	28	0	4.32
36	36	12	12	4	4.32	44	32	8	12	4	4.33
44	24	20	12	0	4.32	44	36	4	12	4	4.33
36	36	8	12	8	4.32	28	20	20	32	0	4.33
40	24	20	12	4	4.32	40	40	8	12	0	4.33
28	36	4	32	0	4.32	28	24	24	24	0	4.33
32	12	28	28	0	4.32	32	32	16	12	8	4.33
36	12	32	20	0	4.32	40	44	4	12	0	4.33
28	24	16	32	0	4.32	44	12	28	16	0	4.33
28	24	24	20	4	4.32	48	28	8	16	0	4.33
28	16	28	24	4	4.32	48	32	4	16	0	4.33
32	24	24	20	0	4.32	32	48	0	12	8	4.33
32	12	32	24	0	4.32	32	20	24	16	8	4.33
36	40	4	12	8	4.32	24	28	16	32	0	4.33
40	16	20	24	0	4.32	24	40	4	32	0	4.34
24	32	12	24	8	4.32	28	40	0	28	4	4.34
28	28	12	28	4	4.32	40	36	12	12	0	4.34
28	32	8	28	4	4.32	40	16	24	16	4	4.34
32	16	24	24	4	4.32	44	28	12	12	4	4.34
36	32	12	12	8	4.32	44	40	0	12	4	4.34
24	36	8	24	8	4.32	32	12	32	20	4	4.34
28	16	28	28	0	4.32	36	24	24	16	0	4.34

32	40	8	12	8	4.32	36	24	24	16	0	4.34
36	16	28	16	4	4.32	48	36	0	16	0	4.34
28	28	20	16	8	4.32	24	24	20	24	8	4.34
36	48	0	12	4	4.32	24	44	0	24	8	4.34
24	24	20	28	4	4.32	36	16	20	28	0	4.34
32	36	12	12	8	4.32	40	48	0	12	0	4.34
48	24	16	12	0	4.32	40	20	24	12	4	4.34
24	28	20	24	4	4.32	44	20	24	12	0	4.34
32	24	24	16	4	4.32	48	24	12	16	0	4.34
32	44	4	12	8	4.32	28	20	20	28	4	4.34
36	8	32	24	0	4.32	36	28	20	12	4	4.34
36	20	16	28	0	4.32	36	12	28	20	4	4.34
36	36	0	28	0	4.32	24	20	24	28	4	4.34
36	32	16	12	4	4.32	28	24	24	16	8	4.34
24	48	0	24	4	4.32	40	12	24	24	0	4.34
24	44	0	28	4	4.32	24	36	12	28	0	4.34
28	36	4	28	4	4.32	24	40	8	28	0	4.34
24	28	16	24	8	4.33	24	24	24	24	4	4.34
24	28	20	20	8	4.33	32	32	8	20	8	4.34
24	48	0	20	8	4.33	48	20	20	12	0	4.34
24	40	4	24	8	4.33	28	20	28	20	4	4.34
28	24	16	28	4	4.33	32	28	12	20	8	4.34
36	44	0	12	8	4.33	24	24	20	32	0	4.35
28	40	0	32	0	4.33	24	24	24	20	8	4.35
24	32	12	32	0	4.33	32	20	28	20	0	4.35
24	36	8	32	0	4.33	32	36	4	20	8	4.35
28	20	24	20	8	4.33	36	24	20	12	8	4.35
44	16	20	20	0	4.33	40	32	16	12	0	4.35
36	28	16	12	8	4.33	40	12	32	16	0	4.35
40	8	32	20	0	4.33	24	32	16	28	0	4.35
32	12	28	24	4	4.35	20	28	20	24	8	4.36
32	28	20	12	8	4.35	28	44	4	12	12	4.36
44	24	16	12	4	4.35	36	4	36	24	0	4.36
20	36	12	24	8	4.35	24	16	28	28	4	4.36
24	44	4	28	0	4.35	28	40	4	16	12	4.36
24	44	0	32	0	4.35	36	20	28	16	0	4.36
32	8	32	28	0	4.35	40	12	28	16	4	4.36
36	32	8	16	8	4.35	20	36	12	28	4	4.37
20	40	8	24	8	4.35	24	20	28	24	4	4.37
24	36	12	16	12	4.35	28	32	8	24	8	4.37
24	40	8	16	12	4.35	44	20	20	12	4	4.37

28	16	24	32	0	4.35	48	44	0	8	0	4.37
28	12	32	24	4	4.35	20	40	8	28	4	4.37
32	20	28	16	4	4.35	24	48	0	16	12	4.37
36	36	4	16	8	4.35	28	20	28	16	8	4.37
28	12	32	28	0	4.35	28	28	12	24	8	4.37
48	20	16	16	0	4.35	32	20	20	20	8	4.37
32	24	16	20	8	4.35	48	16	24	12	0	4.37
36	28	12	16	8	4.35	32	28	8	32	0	4.37
44	12	24	20	0	4.35	32	24	24	12	8	4.37
20	32	16	24	8	4.35	36	20	24	12	8	4.37
36	12	32	16	4	4.35	40	16	28	12	4	4.37
28	16	28	20	8	4.36	40	28	8	20	4	4.37
44	36	8	8	4	4.36	44	8	32	16	0	4.37
44	40	4	8	4	4.36	24	28	20	16	12	4.37
24	44	4	16	12	4.36	28	32	16	12	12	4.37
32	40	0	20	8	4.36	32	24	12	32	0	4.37
36	8	36	20	0	4.36	40	32	4	20	4	4.37
20	44	4	24	8	4.36	44	16	28	12	0	4.37
24	20	24	24	8	4.36	20	32	16	28	4	4.37
24	32	16	16	12	4.36	20	36	12	20	12	4.37
28	20	28	24	0	4.36	24	20	28	20	8	4.37
32	8	36	24	0	4.36	28	28	16	16	12	4.37
24	28	20	28	0	4.36	40	8	28	24	0	4.37
28	16	24	28	4	4.36	48	32	12	8	0	4.37
28	40	8	12	12	4.36	28	28	16	16	12	4.37
32	16	28	16	8	4.36	40	8	28	24	0	4.37
36	12	24	28	0	4.36	48	32	12	8	0	4.37
36	40	0	16	8	4.36	20	48	0	24	8	4.37
24	48	0	28	0	4.36	20	40	8	20	12	4.37
28	36	8	16	12	4.36	28	36	4	24	8	4.37
44	32	12	8	4	4.36	40	4	36	20	0	4.37
44	44	0	8	4	4.36	48	16	20	16	0	4.37
48	40	4	8	0	4.36	32	32	4	32	0	4.37
28	36	12	12	12	4.36	36	28	8	24	4	4.37
28	32	12	16	12	4.36	40	24	12	20	4	4.37
36	24	24	12	4	4.36	36	40	8	8	8	4.37
36	24	16	16	8	4.36	44	28	16	8	4	4.37
24	20	24	32	0	4.36	24	32	12	20	12	4.37
40	28	20	12	0	4.36	24	36	8	20	12	4.37
48	36	8	8	0	4.36	28	12	28	32	0	4.37
